# Extreme stiffness hyperbolic elastic metamaterial for total transmission subwavelength imaging

**DOI:** 10.1038/srep24026

**Published:** 2016-04-04

**Authors:** Hyuk Lee, Joo Hwan Oh, Hong Min Seung, Seung Hyun Cho, Yoon Young Kim

**Affiliations:** 1 WCU Multiscale Design Division, School of Mechanical and Aerospace Engineering,Seoul National University, 599 Gwanak-ro, Gwanak-gu, Seoul 151-744, Korea; 2 Institute of Advanced Machines and Design, Seoul National University, 599 Gwanak-ro, Gwanak-gu, Seoul 151-744, Korea; 3 Center for Safety Measurement, Korea Research Institute of Standards and Science, 267 Gajeong-ro, Yuseong-gu,Daejon 305-340, Korea

## Abstract

Subwavelength imaging by metamaterials and extended work to pursue total transmission has been successfully demonstrated with electromagnetic and acoustic waves very recently. However, no elastic counterpart has been reported because earlier attempts suffer from considerable loss. Here, for the first time, we realize an elastic hyperbolic metamaterial lens and experimentally show total transmission subwavelength imaging with measured wave field inside the metamaterial lens. The main idea is to compensate for the decreased impedance in the perforated elastic metamaterial by utilizing extreme stiffness, which has not been independently actualized in a continuum elastic medium so far. The fabricated elastic lens is capable of directly transferring subwavelength information from the input to the output boundary. In the experiment, this intriguing phenomenon is confirmed by scanning the elastic structures inside the lens with laser scanning vibrometer. The proposed elastic metamaterial lens will bring forth significant guidelines for ultrasonic imaging techniques.

Pursuing fine quality subwavelength imaging of high transmission and low loss has been a critical issue for several decades. Since perfect lens^1^ was proposed as a means to retain evanescent components, many researches on superlenses involving the anomalous negative refraction and amplification of evanescent waves have been reported^2^,^3^,^4^. Nevertheless, the performance of such methods were limited by transmission losses and inherent limitation of restoring only part of evanescent waves^5^,^6^. As an alternative, anisotropic metamaterials with hyperbolic^7^,^8^,^9^,^10^ or eccentric elliptic equi-frequency contours that can support propagation of high wavevectors (evanescent components) have drawn much attention due to their capability to resolve subwavelength objects and design flexibility. Their equi-frequency contours that stretch out larger than that of a background medium make evanescent waves converted into propagating modes inside the metamaterial and transferred to the other side of the lens, thus preserving subwavelength information. Subwavelength imaging with such metamaterial lenses for electromagnetic^11^,^12^,^13^,^14^,^15^,^16^,^17^,^18^,^19^,^20^, acoustic^21^,^22^, and elastic waves^23^,^24^ has been successfully demonstrated by highly anisotropic characteristics.

On the other hand, several studies to enhance the transmission through the lenses have been reported. Higher optical transmission was realized with the Fabry-Perot resonance mechanisms^25^,^26^,^27^ and the radius-dependent permeability for an impedance-matched condition^28^. In the acoustic counterpart, the zero-mass effect has been implemented^29^,^30^,^31^ to overcome the thickness limitation rising from resonance based lens^32^,^33^ which restricts the device’s thickness to be chosen depending on the operating frequency. Specifically, clamped membranes installed along the slits make the Drude-form resonant state^34^ to realize zero effective mass fulfilling impedance matching condition, thus ensuring complete transmission regardless of the lens’s thickness. As such, material properties were delicately tuned to satisfy the conditions for total transmission.

Researches on elastic metamaterial lens for total transmission, however, have not been performed despite increasing demand for a broad range of applications including non-destructive evaluation and biomedical screening. The main reason is that in an elastic solid, unlike electromagnetic and acoustic metamaterials, coupling of shear, bending, and extensional motion makes it difficult to independently control and realize elastic constitutive parameters. Although recent research on elastic metamaterials with local resonances^35^,^36^,^37^,^38^,^39^ paved a way for achieving unique and anomalous effective properties, such methods still have not succeeded in realizing their specific parameters for continuum media. One of the convenient and efficient ways to fabricate such metamaterials may be to fabricate with a single medium by perforating air holes as recently demonstrated^40^,^41^. Nevertheless, this method inevitably decreases the mass density and stiffness of the metamaterial and eventually disturbs wave transmission due to highly mismatched impedance.

In this article, we propose and experimentally demonstrate total transmission subwavelength imaging with a hyperbolic elastic metamaterial lens. The key idea is to use extreme stiffness realized by the properly-designed unit cells of the metamaterial. It is composed only of a single elastic medium, aluminum with voids. The translational resonance of its local resonators induces an extreme stiffness value for waves propagating along the desired direction. So the extreme stiffness compensates for the decreased effective property (effective mass density), thus making total transmission possible. Although several works to independently control elastic stiffness in the specific direction have been reported^42^,^43^, extreme stiffness realization with continuum media evidenced by experimental demonstration has never been done. In addition to that, negative mass perpendicular to the desired wave propagation direction occurs simultaneously due to the same translational resonance, thus forming hyperbolic dispersion for subwavelength imaging. The theoretical explanations why such parameters are essential to achieve both the hyperbolic dispersion and impedance match condition are given by using our explicit analysis of structural dynamics with an equivalent mass-spring model to simulate our continuum version. As for experimental verification, a slab-like lens composed of 21 × 10 unit cells is fabricated in a base plate. Two subwavelength longitudinal wave sources of 35.48 kHz are generated by magnetostrictive transducers^44^,^45^,^46^,^47^ which are proven to have good wave mode tunability (for predominantly generating the lowest symmetric Lamb wave, which is considered in this work). The transmitted wave at the imaging side of the lens is measured with a laser scanning vibrometer. The obtained results are in a good agreement with numerical simulation. Moreover, wave motions at the inside structures of the lens are measured as well to confirm its feasibility of directly transmitting the subwavelength details. The theoretical and experimental results clearly verify the feasibility for total transmission subwavelength imaging with elastic waves, and the results are expected to offer new design methodology for ultrasonic imaging applications.

## Results

### Analysis on highly anisotropic unit cell

**Figure 1:**
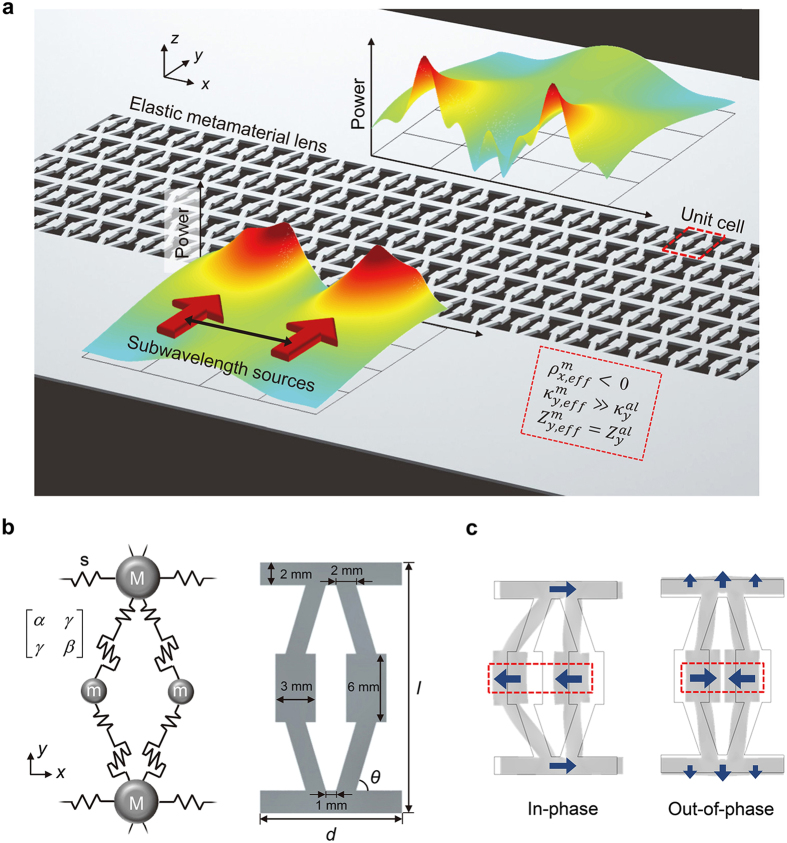
Schematic representation of total transmission of subwavelength imaging with elastic metamaterial lens. (**a**) Concept Schematic of total transmission subwavelength imaging with hyperbolic elastic metamaterial. The constitutive properties of its unit cell are presented in the box with red dashed lines. (**b**) Analytical mass-spring model and its realization in continuum body. (**c**) The two translational resonance eigenmodes: in-phase (left) and out-of-phase (right).

Figure 1a schematically shows the total transmission subwavelength imaging phenomenon with our elastic metamaterial lens. Two subwavelength incident waves (the source width, the distance between the sources, and the center-to-center distances are all of subwavelength scale) impinge perpendicularly in the *y*-direction on the lens, and the unbounded spectrum of spatial frequencies are transmitted to the other side of the lens to form subwavelength images without any loss. The key idea for the complete transmission of subwavelength images is based on satisfying two essential requirements: total transmission condition in a wave propagation direction and a hyperbolically flat dispersion curve over a wide range of transverse wavevectors. At the selected target frequency of 35.48 kHz, we ensure that the parameters of the lens satisfy the following conditions:


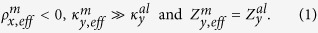


In (1), *ρ*, 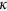, and *Z* denote mass density, stiffness, and impedance, respectively. The indices *m* and *al* stand for metamaterial and aluminum, respectively. The subscript *eff* stands for “effective.” The condition of 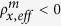 in (1) means that the effective mass density is negative in the *x* direction. Specifically, this single negativity (mass density) in the *x* direction makes a hyperbolic dispersion, similar to negative permittivity/permeability in optical counterparts^12^. In the *y* direction, extremely large stiffness compensates for decreased 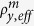 from air perforation, thus fulfilling the impedance matching condition where impedance is 
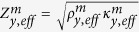
. The extreme stiffness value is vital to total transmission regardless of the lens thickness. This intriguing parametric feature can be obtained with our uniquely designed unit cell shown in Fig. 1b. Both the continuum model and its discrete mass-spring counterpart are shown in the figure.

The fundamental principle of implementing highly anisotropic parameters is to control intrinsic elastic properties stemming from our unique structural configuration. To give an insight into the proposed unit cell, the mass-spring model will be employed. It consists of local resonators consisting of mass *m* connected to the main body mass *M* and a “generalized” spring described by two-dimensional stiffness terms (*α*, *β* and *γ*). The stiffness terms are defined as


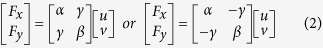


where *u* (*v*) and *F*_*x*_ (*F*_*y*_) are the displacement and force components in the *x* (*y*) direction, respectively. From the definition (2), *α* represents shear stiffness, *β*, extensional stiffness, and the off-diagonal term *γ*, orthogonally coupled stiffness with respect to displacements. (See Supplementary Note for more detailed analysis and discussion). The materialization of the mass-spring model to a continuum body is depicted in the right side of Fig. 1b, where the coupled spring terms are realized as inclined slender beam structures. The whole unit cell is made of aluminum (density *ρ* = 2700 kg/m^3^, Young’s modulus *E* = 70 GPa, and Poisson’s ratio *v* = 0.3). Some of the geometrical dimensions are stated in the figure. The width *d* is 14 mm, the height *l* is 22 mm, and the internal angle of the inclined beam is *θ* = tan^−1^(1/3) = 18.44°.

To reveal the wave characteristics of the metamaterial, it is very useful to retrieve its effective parameters. The use of effective parameters for the characterization is reasonable because the size of the unit is approximately equal to 0.14*λ* in the *y* direction and 0.09*λ* in the *x* direction, thus satisfying the long wavelength metamaterial assumption^48^. Therefore, the effective parameters can be analytically interpreted with our explicit mass-spring model.

The two eigenmodes (in-phase and out-of-phase *x*-directional motions represented with arrows in the red box) of the translational resonance by *m* and (*α*, *β*, *γ*) depicted in Fig. 1c play key roles in bringing about the aforementioned desired extreme values. In this particular case, it should be noted that out-of-phase motion is mainly responsible for the extreme stiffness value in the *y* direction whereas in-phase motion is responsible for negative effective density in the *x* direction. Let us start with verification for out-of-phase motion. Due to orthogonally coupled stiffness terms (by the inclination in the continuum unit cell), out-of-phase motion induces significant deformation in the *y* direction, making an extreme stiffness value in the *y* direction but merely affects the effective density parameter as expressed below in Eqs. (3) and (4). Specifically, the deformation of the unit cell induced by out-of-phase motion exerts a reaction force against the wave propagating motion in the *y* direction, thus giving rise to an extreme stiffness value. Therefore, the effective density (*ρ*_*eff*_) and stiffness (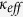) of the unit cell in the *y* direction, of which the detailed analysis is contained in the Supplementary Note, can be expressed as:


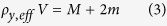



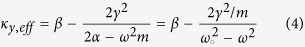


where *V* denotes the volume of the unit cell (i.e., (*ρ*_*y,eff*_*V* = m_*y,eff*_) and 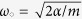 is the translational resonant frequency. This parameter consequently compensates for the decreased impedance in the elastic metamaterial to realize total transmission, which will be explained concretely in the next section.

On the contrary, as for the case of in-phase motion, the translational motion of two resonators in the unit cell cancel out each other’s effect on the deformation in the *y* direction. However, mass density negativity occurs in the *x* direction owing to the dipolar resonance motion of the system consisting of 2*m* and *M*. This phenomenon can be explained otherwise as masses exhibiting a negative total momentum. As discussed above, one can find the effective parameters for the *x* direction (refer to Supplementary Note) as:


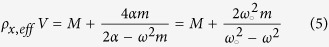



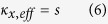


**Figure 2:**
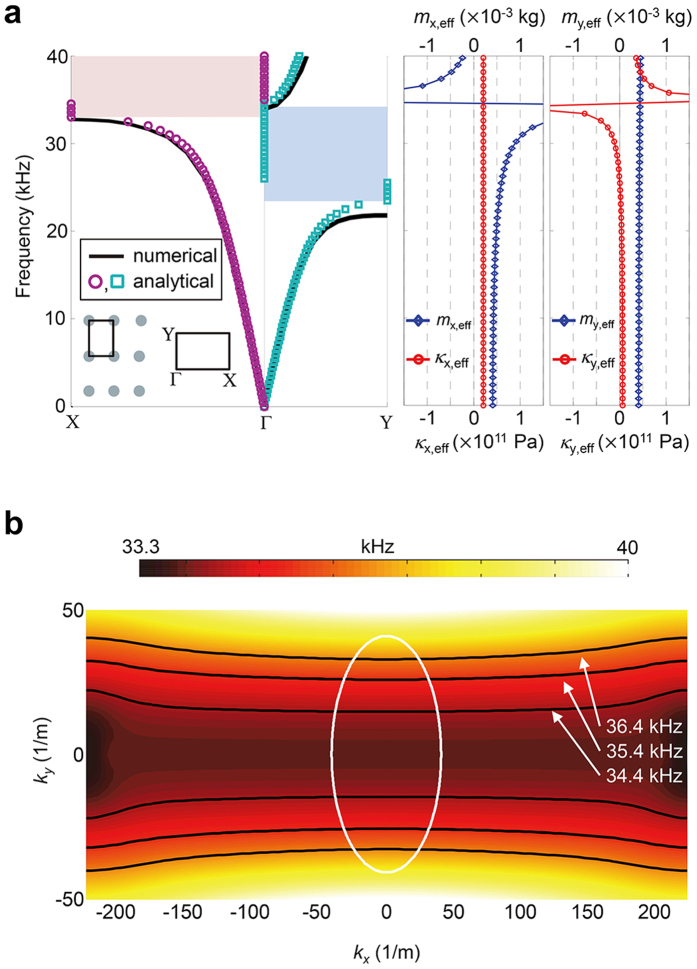
Dispersion relation and effective constitutive properties. (**a**) Dispersion curves for the Г-Χ and Г-Υ directions. Effective properties for both directions are presented in the right. Each negative value induces the resonant bandgap (shaded region), thus causing highly anisotropic properties. (**b**) Equi-frequency contours in a range of frequency. The white contour represents equi-frequency contour of the isotropic aluminum plate at 35.48 kHz.

The band structure of this unit cell is shown in Fig. 2a. In the inset, the rectangular lattice array and the corresponding first Brillouin zone are illustrated. The solid line dispersion curve is numerically calculated by finite element analysis with commercial software COMSOL Multiphysics 3.5a. The circles (for the *x* direction) and squares (for the *y* direction) are obtained by substituting calculated effective material parameters from Eqs. (3, 4, 5, 6) into the following basic periodic mass-spring dispersion relation^49^,


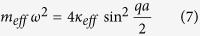


where *q* is the Bloch wave vector and *a,* the periodicity. Dispersion curves retrieved from both methods show a very good agreement. Note that the ranges of the resonant bandgaps in the Г-Χ and Г-Υ directions differ because they rely on different constitutive parameters. Specifically, only the stiffness term experiences a resonant state whereas the mass density term is almost constant in the Г-Υ (i.e., *y*) direction. Exactly the opposite phenomenon occurs in the Г-Χ (i.e., *x*) direction. To realize total transmission in the *y* direction, we elaborately utilize the fact that the effective stiffness based resonant bandgap (light red region) is formed below the resonant frequency *ω*_°_ (*ω*_°_ = 34.64 kHz from the analytical method) whereas the effective mass density based bandgap (light blue region) is formed above the resonant frequency. Therefore, the target frequency for total transmission in the Г-Υ direction is chosen to be 35.48 kHz which is slightly higher than the resonant frequency *ω*_*°*_. In this case, a pass band exists only for the Г-Υ direction while no wave can propagate in the Г-Χ direction because of the resonant bandgap.

In Fig. 2b, we plot the equi-frequency color contours for a range of frequencies around 35.48 kHz. Hyperbolic and nearly flat equi-frequency contours whose widths are several times larger than that of background medium can be clearly seen. It is also observable that such dispersion behavior is valid for a wide range of frequencies. Similar to the canalization mechanism^12^,^17^,^18^,^19^, we can expect our slab-like elastic metamaterial lens to work as a transmission device that allows transportation of high wavevector components from one side to the other. With its hyperbolic dispersion, it enables an unbounded range of wave numbers to be delivered across the lens, ensuring no loss of any imaging information.

More analytical details on manipulating parameters that are necessary to achieve both the hyperbolic dispersion and the impedance matching condition are provided in the Supplementary Note. In fact, although not shown here, unit cells with similar geometries with different parameters were found to possess the desired characteristics, meaning that the unit cell microstructure in Fig. 1b is not the only solution. The main reason for the small discrepancy between the numerical and analytical results is that the discrete mass-spring model cannot totally represent mixture of elastic deformations (bending, torsion, and elongation) that are inherent in the continuum geometry. Nevertheless, the analysis based on the discrete model provides adequate guidelines to analyze and obtain desired parameters.

### Total transmission of lens composed of designed unit cells

**Figure 3:**
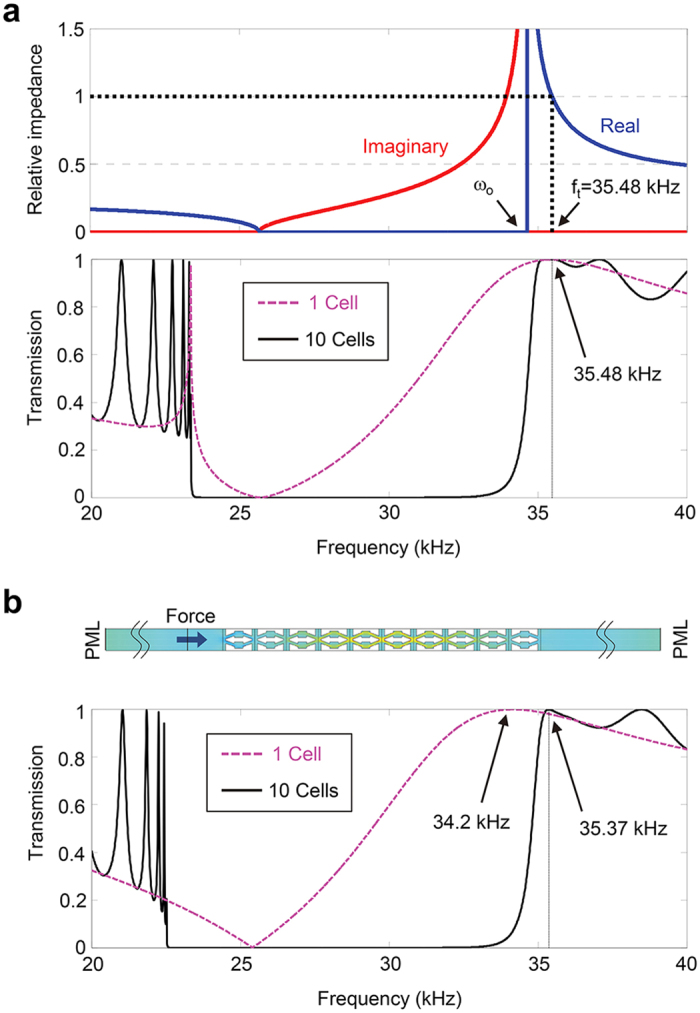
Transmission curve. (**a**) Relative impedance calculated by analytically retrieved properties (upper) and transmission curve (lower). (**b**) Numerical set-up for transmission computation and transmission curve.

In this section, we will show how the total transmission can be realized by using the designed metamaterial lens at the target operating frequency. Figure 3a displays how the relative impedance *Z*_*r,eff*_ varies as a function of frequency along the Г-Υ direction. The relative impedance is defined as 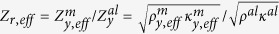 where Eqs. (3, 4) are used for its evaluation. In the negative-stiffness bandgap region below the resonant frequency (*ω*_°_), the relative impedance has only an imaginary part, meaning that no energy propagation is possible in that frequency range. Just above the resonant frequency, the real part of the impedance increases significantly as a result of the stiffness resonant state. The anomalously increased extreme stiffness term can thus counterbalance the reduced effective property (density 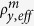) of the metamaterial lens. Slightly above the resonant frequency, the relative impedance is extremely large but it decreases as the frequency further increases. A perfectly-matched impedance (*Z*_*r,eff*_ = 1) occurs at 35.48 kHz.

The one-dimensional transmission coefficient *t* for waves passing through a dissimilar medium can be formulated as


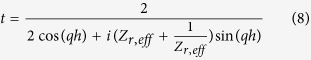


where *h* is the length of a dissimilar medium and in this particular case, *h* represents the thickness of the metamaterial lens inserted in an aluminum medium. The transmission coefficients based on Eq. (8) are plotted in the lower part of Fig. 3a with a different number of unit cells (i.e., 1 and 10 unit cells). The transmission curve indicates that total transmission occurs at 35.48 kHz regardless of the number of unit cells (equivalently, the thickness of the lens). The peak frequency coincides with the impedance matching frequency and is invariant to the change of the number of unit cells. Other observations can be made on additional peaks that occur from the Fabry-Perot resonances. As the number of unit cells increases, or when the lens becomes thicker, additional peaks appear owing to more frequency values satisfying the Fabry-Perot resonance conditions *qh* = *nπ* (*n* is an integer). Correspondingly, more standing waves can be compressed within the thickness of the lens.

To better understand the total-transmission subwavelength-imaging mechanism, some analysis will be useful. When *Z*_*r,eff*_ = 1, condition (8) becomes *t* = 1/[cos (*qh*) + *i* sin (*qh*)] = *e*^*−iqh*^ and the magnitude of *t* becomes unity regardless of *q* (in this case, *k*_*y*_). This implies that any propagating wave in the *y* direction can undergo total transmission. In other words, the above transmission relation is valid over an unlimited range of high transverse wavenumber components, meaning that they all can be converted to propagating waves and be transferred with total transmission.

Further verification of the total transmission is conducted with a continuum model and the simulation set-up is shown in Fig. 3b. A periodic boundary condition is applied to upper and lower sides. Longitudinal plane waves incident along the *x* direction are excited by harmonic forces along the line source at the left side of the metamaterial layer. The transmitted wave profile is picked up at the right side. Perfectly matched layers are added at the left and right end sides to eliminate any reflected waves from boundaries. To facilitate numerical simulations with the continuum model, computations were performed for every 10 Hz. The collected data for the transmitted waves are processed to calculate the transmission coefficient in Fig. 3b by using the transfer-matrix based method^50^. The overall transmission spectrum in Fig. 3b is in very good accordance with that in Fig. 3a. Small discrepancy between the two results is due to local interactions within the continuum unit cell structures. It should be remarked that as in the zero-mass effect in an acoustic regime, the realized total transmission in the elastic regime does not involve the Fabry-Perot resonance which is inappropriate for pulse type ultrasonic inspection.

### Numerical simulation of wave propagation through the metamaterial lens

To confirm the subwavelength resolution imaging capability of our lens, we perform numerical simulations (see the Methods section for more details). As for incident subwavelength wave generation, a 20 mm wide line source (corresponding to 0.13*λ*) is considered. A time-harmonic force of 100 Pa in the *y* direction is applied at the source. It would be worthwhile noting that our lens is designed for in-plane longitudinal waves, and in this specific case with thin plate, the lowest symmetric Lamb wave mode (S_0_) makes a good correspondence. Consequently, the line source mainly generates the S_0_ wave in our simulations because S_0_ has dominant displacement in the *y* direction. The color plot is presented in the absolute value of the *y*-directional normal stress, 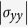. The phase velocity of the S_0_ wave in this condition is 5438 m/s and the corresponding wavelength is 153.6 mm. (For more details on wave characteristics in the media that we consider, refer to Table 1).

**Figure 4:**
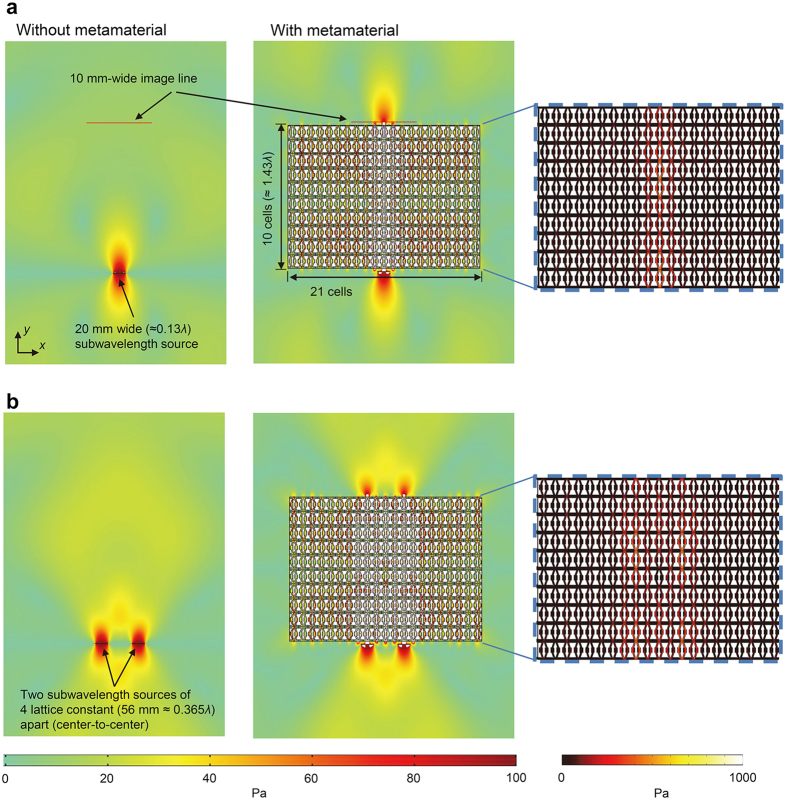
Numerical time-harmonic simulation of subwavelength imaging with elastic metamaterial lens. (**a**) One-source simulation with a 20 mm-wide line source generating the S_0_ wave. The subwavelength information is lost in the far-field without the metamaterial lens whereas it is directly transferred to the other side with the metamaterial. The zoomed plots inside the metamaterial propagation is also presented in the right side. (**b**) Two-source simulation with the same sources separated (center-to-center) by 56 mm is shown. The subwavelength sources are clearly resolved with the metamaterial structure. The color plot is in unit of the absolute *y*-directional stress (|*σ*_*yy*_|) where the input power is 100 Pa in the *y* direction.

With the finite element simulation model, we will verify the subwavelength imaging by the designed metamaterial lens. Figure 4a,b, respectively, consider one-source and two-source cases. For each of the two cases considered, wave simulations with and without the metamaterial lens are carried out. The metamaterial lens is installed 5 mm apart from the source along the *y* direction. From Fig. 4a, it is apparent that in contrast to the case without the metamaterial, the subwavelength details are preserved in the imaging domain when the metamaterial lens is installed as though the source is directly transferred through the lens. The simulation result shows that there is no loss in details for the distance of 10 unit cells (corresponding to 1.43*λ*). In Fig. 4b, two 2 mm-wide line sources with separation of 36 mm (corresponding to 0.23*λ*) and center-to-center separation of 56 mm (corresponding to 0.365*λ*), are installed 5 mm in front of the lens. The two ultrasonic sources separated by a center-to-center distance of 4 lattice constants are clearly resolved after the source signals pass through the metamaterial. Although better resolution can be achieved by narrowing the separation between sources, this setting is chosen for presenting fine quality imaging without side lobes. In fact, two sources as close as 1 lattice constant (14 mm or 0.09*λ*) was resolved under the definition of full-width at half maximum (FWHM) of the peak (shown in Supplementary Fig. S1). In other words, the ultimate resolution is theoretically limited by the period of an unit cell size and thus it can be enhanced with smaller periods^51^.

To visualize wave propagation inside the lens, extended color plotting is provided in the right sides of Fig. 4a,b. Only the regions of wave propagation in the metamaterial lens undergo extremely large deformations, resulting in appreciable stress levels in the regions. These results imply that the system is capable of restricting wave propagation in the *x* direction, which reflects its hyperbolic dispersion characteristics. Additional simulations with different lens thickness, or different unit cell numbers, are presented in Supplementary Fig. S2.

### Experimental verification

**Figure 5:**
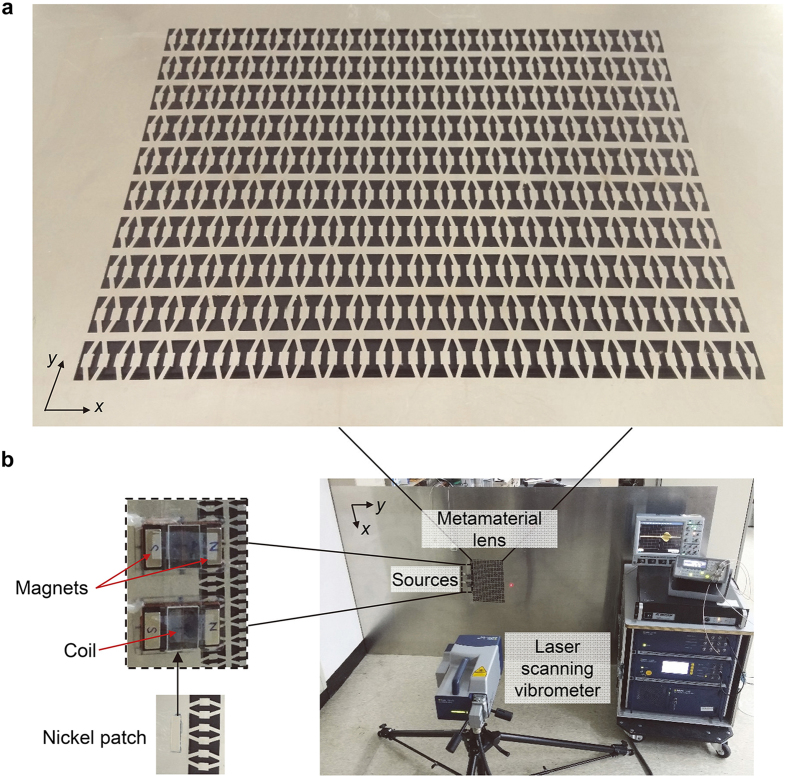
Experimental set-up. (**a**) Fabricated hyperbolic elastic metamaterial lens. It consists of 21 × 10 unit cells. (**b**) Experimental set-up with magnetostrictive transducers and nickel patches that generate the S_0_ wave. The middle of the figure-of-eight coil of the transducer is aligned with the nickel patch. The measurement is carried out by a laser scanning vibrometer.

We experimentally validate the subwavelength imaging phenomenon evidenced with simulation work. In particular, wave motion inside the metamaterial lens is measured. The metamaterial lens shown in Fig. 5a is fabricated in a 1 mm-thick-aluminum plate by a precision laser cutting system. The purpose of small thickness is to comply with the two-dimensional plane stress condition which was implemented for numerical analysis. The dimension of the whole plate is relatively large (2,400 mm × 1,200 mm × 1 mm) to disregard any unwanted reflected waves from its boundaries. The experimental set-up for subwavelength elastic wave imaging is shown in Fig. 5b. The plate where the metamaterial lens is embedded is vertically fixed and ultrasonic magnetostrictive transducers^44^ are installed to generate subwavelength waves. The wave propagation is scanned with a laser scanning head (Polytec PSV-400) that aims perpendicular to the plate surface to measure the normal displacement fields (see more details about generating, measuring, and post processing the ultrasonic waves in the Methods section).

**Figure 6:**
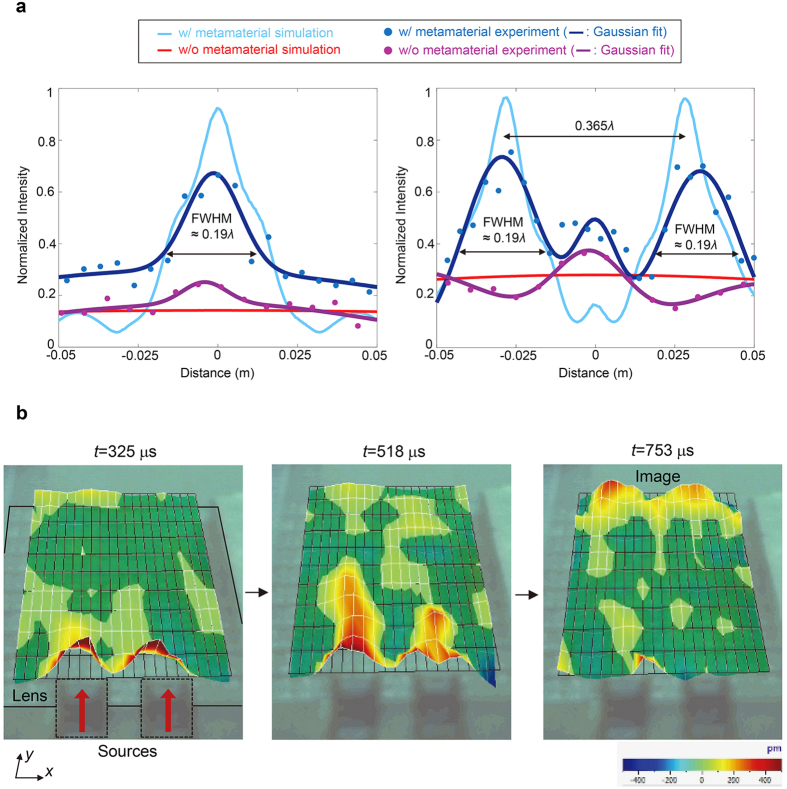
Simulation and experimental results. (**a**) Obtained intensity profiles confirming the subwavelength resolution capability. As for the experimental data, the Gaussian regression method is applied. (**b**) Snapshots from animated images by laser scanning vibrometer software.

The experimental data extracted through fast Fourier Transform (FFT) method are compared with the simulation data in Fig. 6a. Here, the acquired data from simulation are in the unit of *z*-directional normal strain because laser scanning vibrometer measures the displacements normal to the plate surface. The plotted results in Fig. 6 are normalized with respect to the input maximum value from FFT (strain for the simulation plot). The experimentally measured dispacement (as well as strain extracted from simulation) is widely distributed with a small magnitude in the case where there is no metamaterial lens, implying that the subwavelength information is lost in the far-field. In addition, the experimental results are plotted by the Gaussian regression method to characterize the resolution^52^ (more details are presented in the Methods section). The results from the simulation and experiment are in a good agreement. The resolution, by the definition of FWHM, for the one-source experiment is as fine as 29.33 mm or 0.19*λ*. The FWHM’s for two-source experiment is 29.72 mm or 0.19*λ* and 29.93 mm or 0.19*λ*, respectively. Also, the result from two-source experiment shows that peak intensities with subwavelength distance of 56 mm or 0.365*λ* apart can be clearly resolved with the metamaterial lens. The unexpected wave measurement in the middle is attributed to side lobes that are inevitable in experimental setting – we noticed the same behavior with far-field measurement in a bare aluminum without metamaterial.

Because the realization of the subwavelength imaging has been confirmed by the discussions above, we will now analyze the obtained experimental results in terms of total transmission. The peak intensity of single-source case with the metamaterial lens is increased almost by 2.68 times compared to that without one (intensity 0.25 to 0.67). As for the two-source case, the peak intensity is enhanced by 2 and 1.83 times, respectively (intensity 0.37 to 0.74 and 0.68, respectively). It can be clearly seen that the transmitted waves are enhanced with the metamaterial lens compared to those without one. Actually, the exact transmission coefficient must be managed in a one-dimensional point of view. Because waves from two-dimensional line sources can be focused in some spot, measuring the exact transmission coefficient with line sources would not be possible. In fact, if measured closer to the lens, the normalized peak intensity exceeds the unity value. Thus, instead of comparing transmission in two-dimensional point of view, we analyzed it with plane wave assumption in the one-dimensional domain.

One may also question about the fact that the experimentally measured ones with metamaterial lens reach about 70% of the theoretical predictions. Besides inherent material damping, the use of line sources in the experiments not functioning as the ideal line sources in the simulation hinders the realization of the total transmission phenomena. The error can also be attributed to fabrication inaccuracy that can slightly change the resonance frequency, resulting in different effective properties.

On the other hand, an interesting and important experiment is to measure the actual propagation inside the metamaterial lens, considering that no wave measurement through elastic metamaterial lens has been reported. So, additional scanning inside structures of the lens has been carried out as shown in Fig. 6b. The boundaries of the unit cell structures are mainly measured and the mesh grid is as shown in Fig. 6b. The outlines of the lens are also presented. Although the transducers cross the lens boundary, it doesn’t affect the wave generation because the nickel patch (which is installed below the lens) is mainly responsible for transmitting strain to the plate. Therefore, the mesh grid is just above the transducers and stretches up to the output boundary.

In each measurement, iteratively updated signals were automatically averaged over 600 data acquisitions to remove unwanted noise components. Also, a high-pass filter in the laser vibrometer software was used to eliminate high frequency components. The trigger delay was set to 200 μs for experimental convenience. In other words, the source transducers emitted signals at 200 μs. The snapshots were taken from sequential animated images obtained from the laser vibrometer software. From figures in Fig. 6b, one can clearly see that because the group velocity is very low inside the metamaterial lens, the wave propagates to the other side of the lens much slower than the S_0_ wave in an isotropic aluminum plate. In contrast to the group velocity, phase velocity is increased in the metamaterial lens, resulting in elongated wavelength as well. One can observe the positive amplitudes (represented in red) of increased wavelength inside the structure at 518 μs. The details of wave properties for the background aluminum plate and metamaterial lens are presented in Table 1. In addition to that, Fig. 6b clearly shows that the direct propagation of two distinct subwavelength S_0_ waves without spreading.

## Discussion

A hyperbolic elastic metamaterial was proposed and experimented for total transmission in subwavelength imaging by ultrasonic elastic waves. Total transmission was realized by extreme stiffness of the single-medium planar metamaterial lens, controlled by a resonance mechanism in the horizontal translational motion. The unit cell of the metamaterial was elaborately designed to achieve the effective negative mass for horizontally propagating wave motion but the effective extreme stiffness for vertically propagating wave motion at a target frequency. The low effective density in the vertical direction was compensated by the extreme effective stiffness, making the impedance of the metamaterial match with that of the base material. Our explicit analytical method provided how to delicately control the effective elastic parameters that are needed for the hyperbolic dispersion with total transmission. The total transmission phenomenon by the exact impedance matching condition was verified by the experimental results that are consistent with the numerical simulation results. In experiments, the actual propagation inside the metamaterial lens was visualized via laser scanning vibrometer software when full transmission took place.

This planar imaging device has a great potential in practical applications as it is convenient to mount it on a specimen to be inspected. It also increases compatibility when combined with conventional imaging tools. Distinct from imaging devices relying on the Fabry-Perot resonances or thickness resonances, this structure works as an imaging endoscope operating regardless of the lens thickness. Particularly unlike such thickness-relying lenses that some amount of time is required for the stable response from superpositions of waves at the boundaries, this lens allows straightforward imaging which is more suitable in ultrasonic imaging field. To increase the operational bandwidth for total transmission, one may formulate a topology optimization to design the unit cell as in ref. 53. Overall, we expect the results will open doors for advanced ultrasonic imaging techniques with higher performance and practicability.

## Methods

### Finite element numerical simulation

For all the finite element simulations throughout the paper, we utilized the commercial software COMSOL Multiphysics 3.5a installed in a high performance computing cluster. Plane stress, which is appropriate for small thickness, and no damping conditions were considered for all the simulations. The eigenfrequency analysis module was used for the numerically calculated dispersion relation in Fig. 2, whereas the frequency response analysis module was used for other simulations. For the simulation in Fig. 4, the elastic hyperbolic metamaterial lens is composed of 21 × 10 unit cells so that the whole structure is 294 mm wide and 220 mm tall. Perfectly matched layers are applied to entire boundaries to eliminate boundary-reflected waves (not depicted in Fig. 4 for brevity).

### Wave generation in the experiment with magnetostrictive transducers

For proper generation of in-phase S_0_ waves, two magnetostrictive transducers^44^,^45^,^46^ are installed in front of the lens as shown in Fig. 5b. The transducer consists of permanent magnets, a solenoid copper coil array, and a nickel patch that is mainly responsible for wave generation by its magnetostriction phenomenon. Owing to a proper structural assembly of coils and magnets of the employed transducers, they mainly generate the S_0_ mode; dynamic magnetic field induced by the coils is set to be parallel to the static bias field by permanent magnets, causing longitudinal strain for nickel patch along the direction normal to the coils, thus predominantly generating S_0_ waves. Specifically we utilize a figure-of-eight coil to gain good directivity of the magnetic field. (See^44^,^45^,^46^ for more details on the working mechanism of the magnetostrictive transducer).

As shown in Fig. 5b, the nickel patch of the transducer is directly installed on the plate with epoxy bonding, and then the transducer is carefully attached on top of the patch by positioning its coils above the patch. Here, the middle of the figure-of-eight coil is aligned with the nickel patch. For subwavelength sources, the nickel patches are chosen to be 5 mm long (in the *y* direction) and 20 mm wide (in the *x* direction). These patches well mimic two line sources facing normally to the lens that are appropriate for evaluating the capability of subwavelength imaging. Some critical issue exists in that the length (here, 5 mm) of a nickel patch can affect the incident wave properties in some way. When normal strain is induced from magnetostriction, unwanted waves can be simultaneously produced along transverse direction due to Poisson’s ratio effect^46^,^47^. Therefore the shorter the patch size, the less generation of the side waves. However, a tradeoff exists in that the shorter the patch is, the lower intensity power it can produce. We chose it to be 5 mm to minimize the side waves but to guarantee adequate power.

The source signal is emitted from a function generator (Agilent 33220A) and amplified by a low-frequency power amplifier (AE TECHRON 7224) and finally sent to the source transducer. As for two-source experiments, the generation of in-phase source signals is critical. To realize it with magnetostrictive transducers, equal electric current is supplied to each transducer by a serially-connected circuit configuration. To ensure good frequency localization for the sensitive resonant state, we utilize a 20-cycle modulated sine wave  
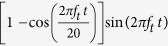
 where *f*_*t*_ is our target frequency 35.48 kHz. Although better frequency localization can be obtained with longer cycles, 20 cycles is chosen to avoid elongation of reflected waves from the plate boundaries.

### Measurement and data processing in the experiment

Regarding the measurement, it should be noted that a laser scanning vibrometer is more suitable for measuring out-of-plane displacements whereas the S_0_ mode has larger in-plane displacements along its propagation direction. However, in-plane displacements of the S_0_ mode can be detected without losing generality thanks to Poisson’s ratio. In this case, one may wonder if anti-symmetric Lamb waves may be dominantly measured instead of the symmetric S_0_ Lamb wave by a vibrometer. Because the actuation frequency is so selected to ensure that the anti-symmetric Lamb wave in this frequency range is in the region of a stop band, its propagation through the metamaterial is forbidden.

Scanning at the output boundary has been conducted to elaborate on its subwavelength resolution ability. The measurements are made in the 100 mm-wide image line that corresponds to the red lines in Fig. 4a. This line is 230 mm apart (in the *y* direction) from the sources, which is 5 mm apart from the output boundary when there is a metamaterial lens. The purpose of measuring close to the metamaterial is to acquire the wave signals before they rapidly decay in the non-metamaterial base aluminum plate. Regarding data processing, filtering only the desired frequency is necessary because the dispersion curve of the metamaterial is very sensitive to the resonant frequency and the input signal contains a certain range of frequency components. Also, the raw data contain unwanted frequency components and noise owing to high sensitivity of the laser scanning vibrometer. Thus, the arrival signals at the output boundary are post-processed by the FFT method.

### Gaussian regression for resolution characterization

To characterize the resolution by deriving the FWHM, triple summations (to consider the case of two-source imaging where two beam profiles and a sidelobe exist) of the Gaussian functions were utilized for regression.


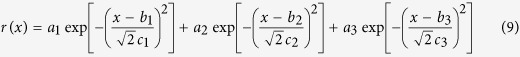


The coefficients *a*, *b*, and *c*’s for each case ensure the curves to fit the data with the R^2^ (coefficient of determination) value, the goodness of fit, to exceed 80%.

## Additional Information

**How to cite this article**: Lee, H. *et al.* Extreme stiffness hyperbolic elastic metamaterial for total transmission subwavelength imaging. *Sci. Rep.* **6**, 24026; doi: 10.1038/srep24026 (2016).

## Supplementary Material

Supplementary Information

## Figures and Tables

**Table 1:** Wave properties in the wave propagation direction (*y* direction) of the two media.

Wave properties	1 mm-thick aluminum plate	Metamaterial lens
Group velocity (m/s)	5407	792
Phase velocity (m/s)	5438	8669
Wavelength (mm)	153.6	244.3
