# Supplementary material for: Extreme stiffness hyperbolic elastic metamaterial for total transmission subwavelength imaging
Source: Sci Rep. 2016 Apr 4;6:24026. doi: 10.1038/srep24026 (PMC4819191; doi:10.1038/srep24026)
Supplement: Supplementary Information [file srep24026-s1.pdf]

Extreme stiffness hyperbolic elastic metamaterial for total transmission subwavelength imaging

Hyuk Lee[1], Joo Hwan Oh[2], Hong Min Seung[1], Seung Hyun Cho[3], and Yoon Young Kim[1,2,*]

[1]*WCU Multiscale Design Division, School of Mechanical and Aerospace Engineering, Seoul National University, 599 Gwanak-ro, Gwanak-gu, Seoul 151-744, Korea*

[2]*Institute of Advanced Machines and Design, Seoul National University, 599 Gwanak-ro, Gwanak-gu, Seoul 151-744, Korea*

[3]*Korea Research Institute of Standards and Science, 267 Gajeong-ro, Yuseong-gu, Daejon 305-340, Korea*

Supplementary Note

Analytic modelling

Our unit cell system can be analytically explained with a mass-spring model as shown in Fig. S1 (a). As a unit cell, we choose the mass-spring system enclosed in the red box shown in Fig. S1 (b). The mass *M* is considered to be connected with mass *m*. Therefore, one can model this system by an equivalent system consisting of effective mass and stiffness in *x* and *y* directions as suggested at the right side of Fig. S1 (b). The horizontal and vertical displacements of mass *M* at the (*i*,*j*)th unit cell are denoted by $U_{i,j}$ and $V_{i,j}$, respectively. The mass *M* is connected to the mass in the adjacent unit cell by spring *s*, and local resonators by coupled springs as illustrated in the figure. The coefficients (α, β, γ) of the coupled spring can be defined as

* Corresponding Author, Professor, yykim@snu.ac.kr, phone +82-2-880-7154, fax +82-2-872-1513

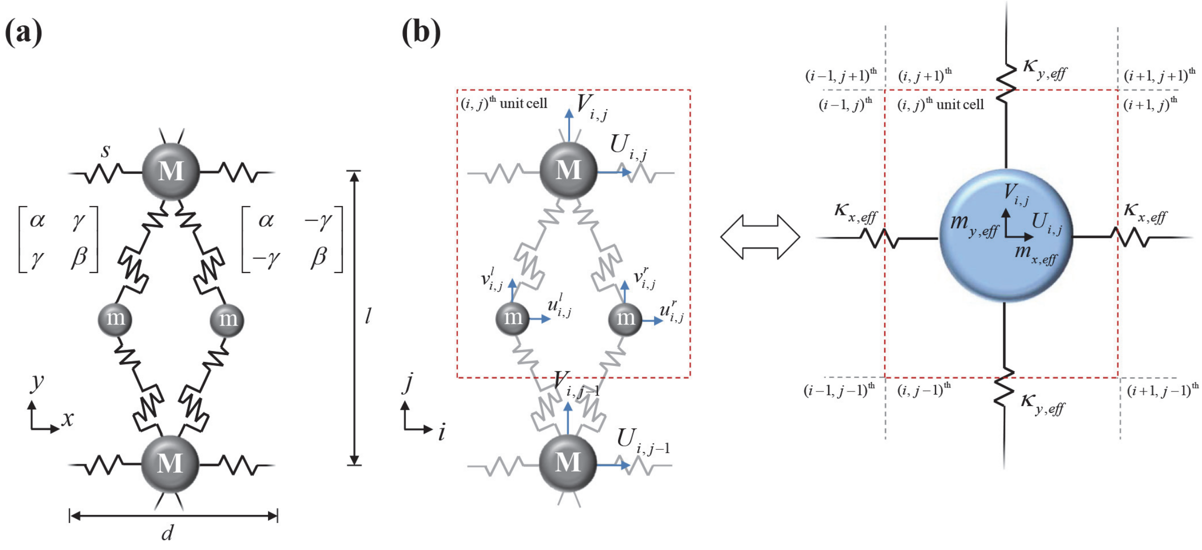

Fig. S1. (a) Mass-spring model of unit cell configuration. (b) Displacement notations and a set of masses to be considered as a new unit cell for deriving the parameters. Its effective mass and stiffness terms are equivalently represented in a simple mass-spring system in the right.

$$\begin{bmatrix} F_x \\ F_y \end{bmatrix} = \begin{bmatrix} \alpha & \gamma \\ \gamma & \beta \end{bmatrix} \begin{bmatrix} u \\ v \end{bmatrix} \text{ or } \begin{bmatrix} F_x \\ F_y \end{bmatrix} = \begin{bmatrix} \alpha & -\gamma \\ -\gamma & \beta \end{bmatrix} \begin{bmatrix} u \\ v \end{bmatrix}. \tag{S1}$$

where F_i is the applied force in the *i* (*i*=*x*,*y*) direction and the displacement components of mass *m* in the *x*, *y* directions are denoted by *u* and *v*. Stiffness α represents shear stiffness while β, normal stiffness. The off-diagonal term γ is the coupling stiffness appearing due to the inclination of the elastic medium connecting *M* and *m*. The displacement variables of two resonator masses are denoted by $(u^l_{i,j}, v^l_{i,j})$ and $(u^r_{i,j}, v^r_{i,j})$.

Basic equation for one-dimensional simple mass-spring system

To show briefly how the dispersion relation and effective parameters are retrieved, we consider only the *x*-directional components in the right figure of Fig. S1 (b). The equilibrium equation of the $(i,j)^{\text{th}}$ mass in the *x* direction can be expressed in terms of the *x*-directional

displacement $U_{i,j}$ as

$$m_{x,eff}\frac{\partial^2 U_{i,j}}{\partial t^2} = \kappa_{x,eff}(U_{i+1,j} - U_{i,j}) - \kappa_{x,eff}(U_{i-1,j} - U_{i,j}). \quad \text{(S2)}$$

where $\kappa_{x,eff}$ is the effective stiffness in the *x* direction. Assuming time harmonic wave motion at an angular frequency of ω, the displacement $U_{i,j}$ is re-written as $U_{i,j}\exp[i(\omega t - k_x x)]$ where *k* is the wavenumber. By using $\partial^2 U_{i,j}/\partial t^2 = -\omega^2 U_{i,j}$, $U_{i+1,j} = \exp(-ik_x d)U_{i,j}$, and $U_{i-1,j} = \exp(ik_x d)U_{i,j}$ the dispersion relation can be expressed as

$$-\omega^2 m_{x,eff} = \kappa_{x,eff}(\exp(-ik_x d) + \exp(ik_x d) - 2). \quad \text{(S3)}$$

This is the basic form of simple periodic mass-spring relation and will be applied in this work to derive the effective parameters.

x-directional parameters: resonant mass density

Let us first derive the parameters in the *x* direction. Because we consider only longitudinal wave motion, the vertical displacements of *M*, $V_{i,j}$, will be omitted. Also, from the symmetric condition, $v^l_{i,j} = v^r_{i,j} = 0$. Thus, the equations of motion for the mass components in the $(i,j)^{\text{th}}$ cell become

$$M\frac{\partial^2 U_{i,j}}{\partial t^2} = s(U_{i+1,j} + U_{i-1,j} - 2U_{i,j}) + \alpha(u^l_{i,j} + u^r_{i,j} + u^l_{i,j+1} + u^r_{i,j+1} - 4U_{i,j}), \quad \text{(S4a)}$$

$$m\frac{\partial^2 u^l_{i,j}}{\partial t^2} = \alpha(U_{i,j} + U_{i,j-1} - 2u^l_{i,j}), \quad \text{(S4b)}$$

$$m\frac{\partial^2 u^r_{i,j}}{\partial t^2} = \alpha(U_{i,j} + U_{i,j-1} - 2u^r_{i,j}). \quad \text{(S4c)}$$

For longitudinal motion in the *x* direction, the displacements can be assumed not to vary along the *y* direction. In this case, the following conditions are satisfied:

$$U_{i,j+1} = U_{i,j} = U_{i,j-1}, \tag{S5a}$$

$$u_{i,j}^{l} = u_{i,j+1}^{l}, \tag{S5b}$$

$$u_{i,j}^{r} = u_{i,j+1}^{r}. \tag{S5c}$$

For steady state time-harmonic wave motion at an angular frequency of ω, and conditions (S5a-c), equations (S4a) to (S4c) reduce to

$$-\omega^2 M U_{i,j} = s(\exp(-ik_x d) + \exp(ik_x d) - 2)U_{i,j} + 2\alpha(u_{i,j}^{l} + u_{i,j}^{r} - 2U_{i,j}), \tag{S6a}$$

$$-\omega^2 m u_{i,j}^{l} = 2\alpha(U_{i,j} - u_{i,j}^{l}), \tag{S6b}$$

$$-\omega^2 m u_{i,j}^{r} = 2\alpha(U_{i,j} - u_{i,j}^{r}). \tag{S6c}$$

Equation (S6b) and (S6c) can be re-written as

$$u_{i,j}^{l} = u_{i,j}^{r} = \frac{2\alpha}{2\alpha - \omega^2 m} U_{i,j}. \tag{S7}$$

Substituting equation (S7) into (S6a) yields

$$-\omega^2 M U_{i,j} = s(\exp(-ik_x d) + \exp(ik_x d) - 2)U_{i,j} + \frac{4\alpha\omega^2 m}{2\alpha - \omega^2 m} U_{i,j}. \tag{S8}$$

By re-writing this equation in the form of basic simple mass-spring dispersion relation, the following equation can be obtained

$$-\omega^2 (M + \frac{4\alpha m}{2\alpha - \omega^2 m}) = s(\exp(-ik_x d) + \exp(ik_x d) - 2). \tag{S9}$$

Comparing this with (S3), the effective parameters can be defined as

$$m_{x,eff} = M + \frac{4\alpha m}{2\alpha - \omega^2 m} = M + \frac{2\omega_\circ^2 m}{\omega_\circ^2 - \omega^2}, \tag{S10a}$$

$$\kappa_{x,eff} = s, \tag{S10b}$$

where $\omega_\circ = \sqrt{2\alpha / m}$ is the x-directional resonant frequency. Therefore, only the mass term becomes the function of ω whereas the effective stiffness is independent of ω.

***y*-directional parameters: resonant stiffness**

As for the y direction, a similar approach will be made. Here, the only difference is that the x-directional motion of resonators is always coupled with the y-directional motion. Thus, the equations of motion for mass components in the $(i,j)^{\text{th}}$ cell can be written as follows:

$$M\frac{\partial^2 V_{i,j}}{\partial t^2} = \beta(v^l_{i,j} + v^r_{i,j} + v^l_{i,j+1} + v^r_{i,j+1} - 4V_{i,j}) + \gamma(u^l_{i,j} - u^r_{i,j} - u^l_{i,j+1} + u^r_{i,j+1}), \tag{S11a}$$

$$m\frac{\partial^2 v^l_{i,j}}{\partial t^2} = \beta(V_{i,j} + V_{i,j-1} - 2v^l_{i,j}), \tag{S11b}$$

$$m\frac{\partial^2 v^r_{i,j}}{\partial t^2} = \beta(V_{i,j} + V_{i,j-1} - 2v^r_{i,j}), \tag{S11c}$$

$$m\frac{\partial^2 u^l_{i,j}}{\partial t^2} = \gamma V_{i,j} - \gamma V_{i,j-1} - 2\alpha u^l_{i,j}, \tag{S11d}$$

$$m\frac{\partial^2 u^r_{i,j}}{\partial t^2} = -\gamma V_{i,j} + \gamma V_{i,j-1} - 2\alpha u^r_{i,j}. \tag{S11e}$$

Assuming time-harmonic wave motion, $V_{i,j} = V_{i,j}\exp[i(\omega t - k_y y)]$ and $V_{i,j-1} = \exp(ik_y y)V_{i,j}$, equation (S11) becomes

$$\begin{aligned}-\omega^2 MV_{i,j} = -4\beta V_{i,j} + \beta(1+\exp(-ik_y l))v^l_{i,j} + \beta(1+\exp(-ik_y l))v^r_{i,j} \\ + \gamma(1-\exp(-ik_y l))u^l_{i,j} - \gamma(1-\exp(-ik_y l))u^r_{i,j}\end{aligned}, \tag{S12a}$$

$$-\omega^2 mv^l_{i,j} = \beta(1+\exp(ik_y l))V_{i,j} - 2\beta v^l_{i,j}, \tag{S12b}$$

$$-\omega^2 mv^r_{i,j} = \beta(1+\exp(ik_y l))V_{i,j} - 2\beta v^r_{i,j}, \tag{S12c}$$

$$-\omega^2 mu^l_{i,j} = \gamma(1-\exp(ik_y l))V_{i,j} - 2\alpha u^l_{i,j}, \tag{S12d}$$

$$-\omega^2 mu^r_{i,j} = -\gamma(1-\exp(ik_y l))V_{i,j} - 2\alpha u^r_{i,j}. \tag{S12e}$$

Re-writing equations (S12b-e) with respect to $V_{i,j}$ yields

$$v_{i,j}^{l} = v_{i,j}^{r} = \frac{\beta(1+\exp(ik_y l))}{2\beta-\omega^2 m} V_{i,j} \tag{S13a}$$

$$u_{i,j}^{l} = -u_{i,j}^{r} = \frac{\gamma(1-\exp(ik_y l))}{2\alpha-\omega^2 m} V_{i,j} \tag{S13b}$$

By substituting equation (S13a-b) into (S12a), the dispersion relation for the y direction can be obtained as

$$-\omega^2 (M + \frac{4\beta m}{2\beta-\omega^2 m}) = \left[\frac{2\beta^2}{2\beta-\omega^2 m} - \frac{2\gamma^2}{2\alpha-\omega^2 m}\right](\exp(-ik_y l)+\exp(ik_y l)-2) \tag{S14}$$

Here, it must be noted that in contrast to the former case, not only the effective mass but also stiffness terms are functions of ω. As equation (S14) shows, the y-directional resonance occurs mainly due to the β term. For the selected geometry (d=14 mm, l=22 mm), one can identify m, M, α, β, and γ as those shown in Table S1. As shown in Table S1, $\beta \gg \alpha$ so that the y-directional resonance occurs at a much higher frequency than that for the x-directional resonance. Because α is related to shear modulus and β, to tension, β is usually much larger than α. However, in a special situation when the operating frequency is much lower than the y-directional resonant frequency $\omega_\circ' = \sqrt{2\beta / m}$, one can assume that

$$2\beta - \omega^2 m \approx 2\beta. \tag{S15}$$

If the assumption (S15) is valid, equation (S14) reduces to

$$-\omega^2 (M+2m) = \left[\beta - \frac{2\gamma^2}{2\alpha-\omega^2 m}\right](\exp(-ikl)+\exp(ikl)-2) \tag{S16}$$

Consequently, the effective mass and stiffness for the y direction become

$$m_{y,eff} = M + 2m, \tag{S17a}$$

$$\kappa_{y,eff} = \beta - \frac{2\gamma^2}{2\alpha-\omega^2 m} = \beta - \frac{2\gamma^2 / m}{\omega_\circ^2 - \omega^2}. \tag{S17b}$$

α	β	γ	M	m
1.87 GPa	12.69 GPa	3.41 GPa	2.53e-4 kg	7.89e-5 kg

Table. S1. The calculated values for mass and stiffness parts of the analytical mass-spring model from the continuum unit cell. The calculations were performed by the finite element method with COMSOL Multiphysics 3.5a.

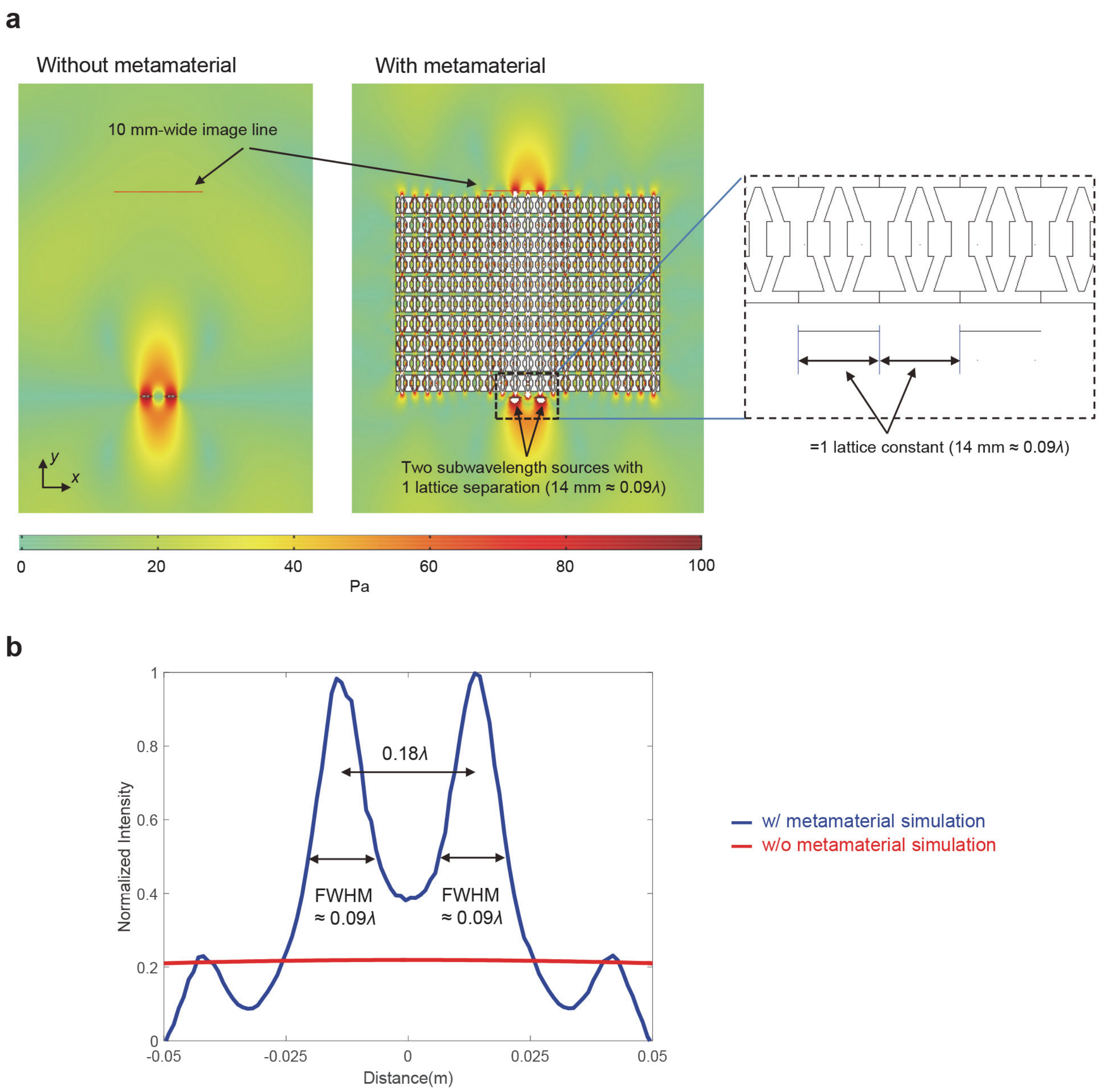

Fig. S1. (a) Numerical simulation for two sources as close as 1 lattice constant (14 mm or 0.09λ). The specific parameters are presented in the right side. All the simulation set-up and condition were the same as those stated in the manuscript. **(b)** Obtained intensity profiles conforming the subwavelength imaging capability.

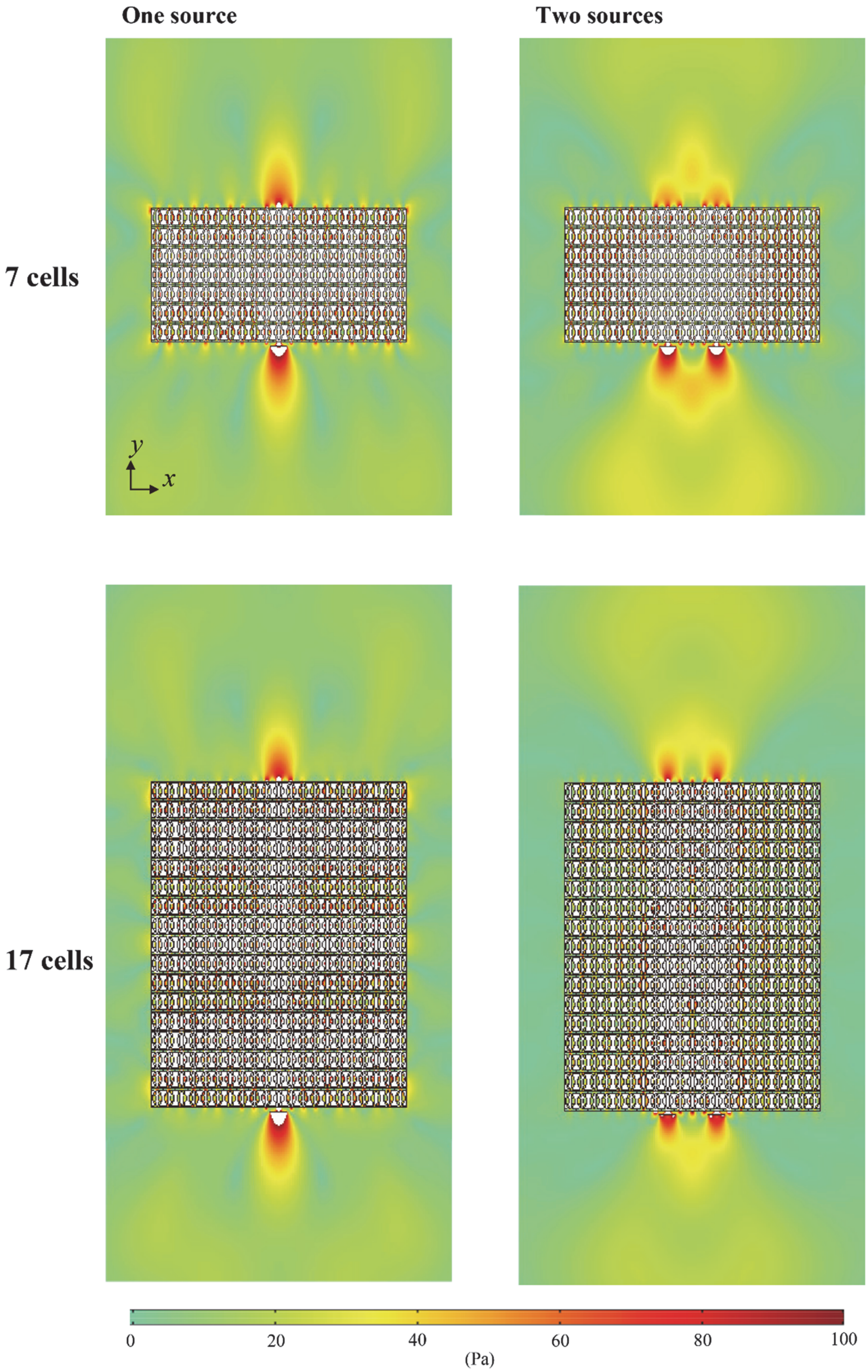

Fig. S2. Additional simulations for different thicknesses (not belonging to the Fabry-Perot resonance or finite resonance) of elastic metamaterial lenses. Thicknesses of 7 cells ($\approx 1\lambda$) and 17 cells ($\approx 2.43\lambda$) are considered. The resolved images are clearly observed in all figures.

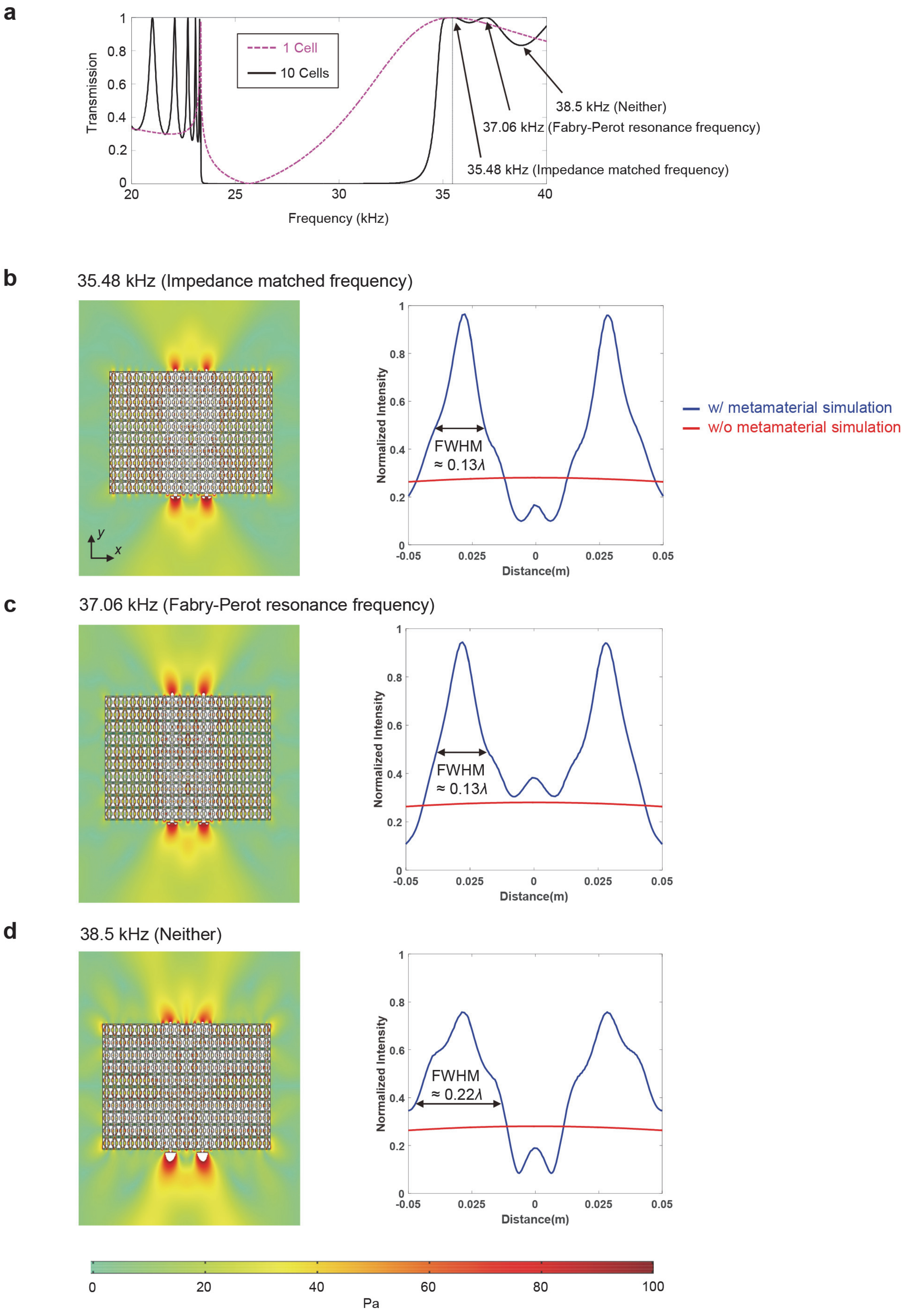
a
Transmission
Frequency (kHz)
1 Cell
10 Cells
38.5 kHz (Neither)
37.06 kHz (Fabry-Perot resonance frequency)
35.48 kHz (Impedance matched frequency)
b
35.48 kHz (Impedance matched frequency)
y
x
Normalized Intensity
Distance(m)
FWHM
≈ 0.13λ
w/ metamaterial simulation
w/o metamaterial simulation
c
37.06 kHz (Fabry-Perot resonance frequency)
FWHM
≈ 0.13λ
d
38.5 kHz (Neither)
FWHM
≈ 0.22λ
Pa

Fig. S3. (a) Transmission curve denoting the three cases (impedance matched case, Fabry-Perot resonance case, and neither one) to confirm the subwavelength resolution capability by numerical simulation. **(b)** Impedance matched case. **(c)** Fabry-Perot resonance case. **(d)** Neither case. Here, it must be noted that all three cases belong to the hyperbolic dispersion range as shown in Fig. 2b in the manuscript. In other words, all three cases are capable of resolving subwavelength sources, although the image quality drops when transmission is not adequate.

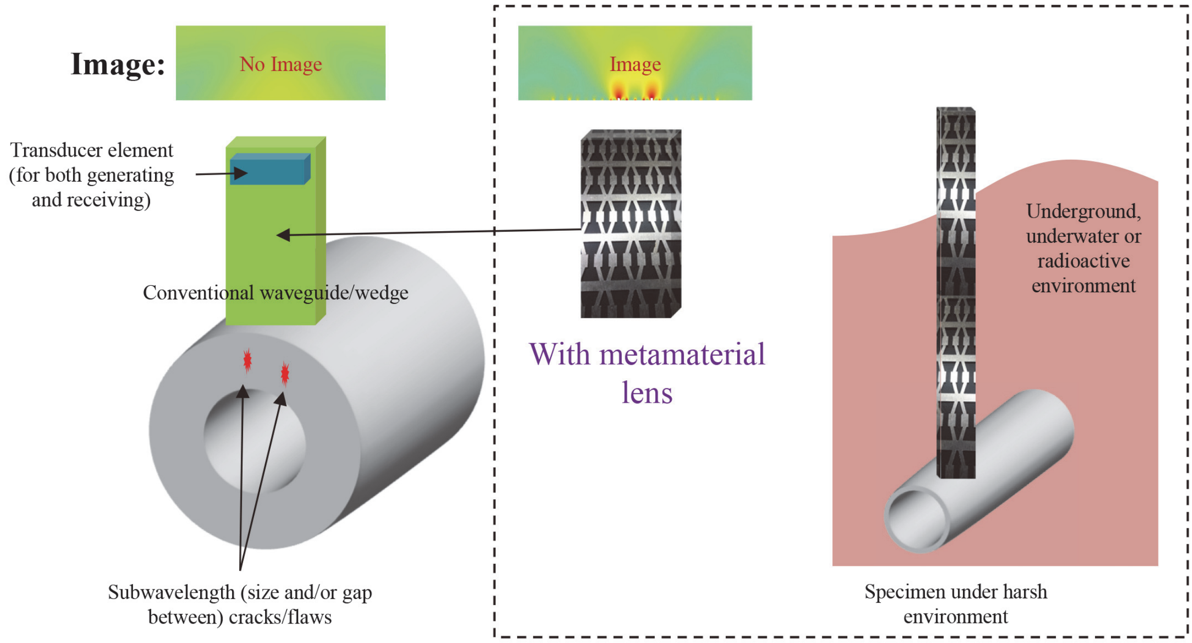

Fig. S4. Concept drawing for practical applications in the ultrasonic imaging technique. By integrating our metamaterial lens into a conventional non-destructive imaging system, we expect to find hidden cracks/flaws that are under subwavelength scale. Because our metamaterial lens promises good transmission and works as an endoscope, it could act as a waveguide that are useful under harsh environments (where pipes are buried underwater or deep down the earth or in radioactive surroundings).
